# Chlorogenic Acid and Rutin Play a Major Role in the *In Vivo* Anti-Diabetic Activity of *Morus alba* Leaf Extract on Type II Diabetic Rats

**DOI:** 10.1371/journal.pone.0050619

**Published:** 2012-11-21

**Authors:** Attila Hunyadi, Ana Martins, Tusty-Jiuan Hsieh, Adrienn Seres, István Zupkó

**Affiliations:** 1 Institute of Pharmacognosy, University of Szeged, Szeged, Hungary; 2 COST Action CM0804 (Chemical Biology with Natural Products) of the European Commission, Brussels, Belgium; 3 Grupo de Micobactérias, Unidade de Parasitologia e Microbiologia Medica, Instituto de Higiene e Medicina Tropical, Universidade Nova de Lisboa, Portugal; 4 Department of Medical Microbiology and Immunobiology, Faculty of Medicine, University of Szeged, Szeged, Hungary; 5 Department of Medical Genetics, Kaohsiung Medical University, Kaohsiung, Taiwan; 6 Department of Pharmacodynamics and Biopharmacy, University of Szeged, Szeged, Hungary; Broad Institute of Harvard and MIT, United States of America

## Abstract

The leaves of the white mulberry tree (*Morus alba* L.) are used worldwide in traditional medicine as anti-diabetics. Various constituents of mulberry leaves, such as iminosugars (*i.e.* 1-deoxynojirimicin), flavonoids and related compounds, polysaccharides, glycopeptides and ecdysteroids, have been reported to exert anti-diabetic activity, but knowledge about their contribution to the overall activity is limited. The objective of the present work was to determine the *in vivo* anti-diabetic activity of an extract of mulberry leaves (MA), and to examine to what extent three major constituents, chlorogenic acid, rutin and isoquercitrin, might contribute to the observed activity. Quantities of the three constituents of interest in the extract were determined by using HPLC-DAD. Activity was determined by using a type II diabetic rat model. After 11 days of *per os* administration of 250 or 750 mg/kg of MA or the corresponding amounts of each individual compound, a dose dependent decrease of non-fasting blood glucose levels were found for MA, chlorogenic acid and rutin, but not for isoquercitrin. Based on our results, chlorogenic acid and rutin might account for as much as half the observed anti-diabetic activity of MA, hence they can be considered as excellent markers for the quality control of mulberry products.

## Introduction

In the last decades, diabetes has become a major health problem both in developed and in developing countries. Moreover, projections for its prevalence seem to be less and less optimistic. In 2000 there were around 171 million of diabetic people and an increase to 366 million was estimated for 2030 [1]. Data from 2010, however, showed that prevalence increased to *ca.* 285 million and 439 million affected adults are now expected till 2030 [2]. Type 2 diabetes mellitus is responsible for over 90% of the overall cases. Treatment of this form of the disorder involves changes in lifestyle and the use of oral antidiabetics [3], which, as it can be seen from the rapidly growing number of affected people, is far from being the solution. Novel therapeutic options have to be considered, including the use of phytotherapeutics as alternative or complementary agents which may crucially improve the life quality of the patients.

The leaves and root bark of the white mulberry tree (*Morus alba* L.) are known worldwide as sources of phytotherapeutics, which have traditionally been used for the treatment of conditions related to type II diabetes. A large number of *in vivo* animal [4]–[9] and human studies [10]–[13] supported the anti-diabetic activity of various mulberry preparations. Many different types of constituents have been linked to the anti-diabetic activity of mulberry drugs, according to the followings:

1. Several iminosugars (or piperidine alkaloids) including 1-deoxynojirimycin were isolated from mulberry leaves, root bark and fruits [14]–[16], and found to inhibit α-amilase and galactosidase. Among these iminosugars, nojirimycin derivatives were the most effective ones. In fact, Miglitol (Diastabol®), an oral antidiabetic drug in Europe, is a synthetic derivative of 1-deoxynojirimycin, and both Acarbose (Glucobay®) and Voglibose (Basen®) show similarity to its structure.
2. Flavonoids and related constituents found in mulberry species were also described for their anti-diabetes-related effects: a concentrated flavonoid fraction from the root bark of *M. alba* exerted protective effect on rat pancreatic β-cells against streptozotocin (STZ) [17], and the leaves of a related South-American mulberry species, *M. insignis*, were found to contain two benzofurane derivatives that were active on STZ induced diabetic rats [18]. On the other hand, a partially purified flavone fraction of *M. alba* was found to activate α-glucosidase enzyme, which might lead to an increase in the blood glucose level [19].
3. Polysaccharides from *M. alba* leaves exerted strong competitive inhibition of α-glucosidase [19].
4. Glycopeptides isolated from the root bark of the white mulberry tree showed insulin-like effect on experimentally induced diabetic rats [4], [7].
5. An apolar, volatile-oil like fraction of a hot water extract (*i.e.* tea) of *M. alba* leaves was found to increase the *in vitro* glucose consumption of adipocytes, and several phenyl-propane derivatives including cinnamic acid were identified in the fraction by means of GC-MS [20].
6. Ecdysteroids (20-hydroxyecdysone and inokosterone [21]) might also contribute to the anti-diabetic activity of mulberry leaves, as these compounds can enhance tissue sensitivity to insulin in rats when applied *per os* [22].

**Figure 1:**
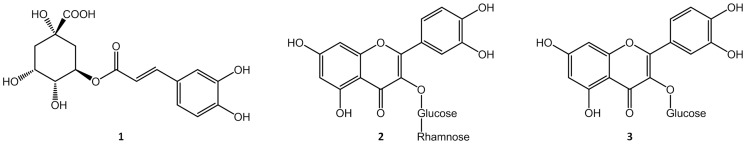
Structures of chlorogenic acid (1), rutin (2) and isoquercitrin (3).

Despite (or exactly due to) the richness of *M. alba* leaves in various constituents exerting anti-diabetic activity, an overview on the relative importance of the individual compounds regarding the complex anti-diabetic effect is not available in the literature. However, such evaluation would be very important for a rational standardization of mulberry products. In view of the scientific literature, currently the iminosugars seem to be held the most important active constituents of mulberry leaves. These compounds lack a UV absorbing cromophore group, which makes them less convenient marker compounds: most typically sophisticated HPLC-MS techniques are suggested to their determination [23], [24].In our preliminary experiments [25], we have found high amounts of chlorogenic acid, rutin and isoquercitrin present in a 70% ethanol extract of the white mulberry leaves, which extract was active *in vivo*; structures of these three compounds are shown in Figure 1.

Considering that 10 mg/kg of chlorogenic acid was previously found to exert significant hypoglycaemic activity on non-neonatal streptozotocin induced diabetic rats [26] and rutin has also been described for its hypoglycaemic activity at a dose of 25 mg/kg on such a rat model [27], these compounds might significantly contribute to the overall anti-diabetic action of the extract. Results of the investigation of this hypothesis are described and discussed in the present paper.

## Results and Discussion

STZ is a natural substance specifically toxic to pancreatic β cells. For this reason it is widely utilized to induce diabetes in mice and rats. The characteristics of the developing diabetes are fundamentally determined by the age of the animal. A single or repeated treatment of adult rats results in a sharply and seriously elevated plasma glucose level accompanied by polyuria and polydipsia, and the correction of hyperglycemia requires parenteral insulin treatment. This state shares the characteristics of insulin-dependent diabetes mellitus (IDDM) and is therefore the intervention of choice when hyperglycemia-induced structural or functional deviations are to be studied [28]. Although the high plasma glucose level is hard to be modified by orally administered agents, this state (IDDM) is frequently but inappropriately considered as an experimental model of non-insulin dependent diabetes mellitus (NIDDM).

On the other hand, NIDDM can be elicited by a single intraperitoneal STZ treatment of neonatal rats [29], [30]. At 8–10 weeks of age and thereafter, rats show nearly normal fasting but substantially elevated postprandial blood glucose levels. It is important to mention, that due to the nearly normal fasting blood glucose levels, the effect on the postprandial blood glucose levels can only be utilized as a measure of bioactivity in this model. Although this certainly introduces the variable of food intake that can influence the results, the overall conditions are more similar to the situation in humans, and, as a practical aspect, the design of fasting periods during a long-term experiment also becomes unnecessary. Therefore, this approach provides a convenient model of NIDDM [31], and it was used in the present study. Moreover, our preliminary experiments also showed that the 70% ethanol extract of mulberry leaves is active on this model [25].

**Figure 2:**
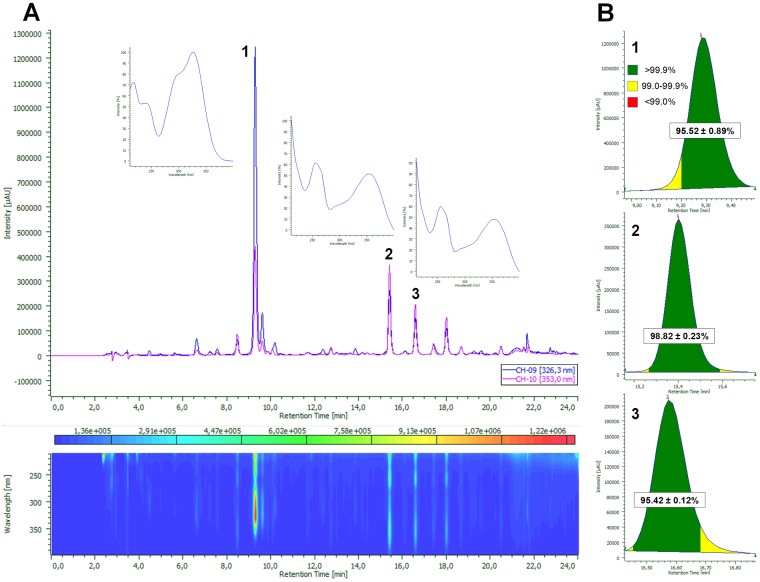
DAD fingerprint of an analyzed sample of mulberry leaf extract together with the chromatograms at λ = 326.3 nm (used for determination of 1) and λ = 353.0 nm (used for determination of 2 and 3) and UV spectra of each compound (Fig. 2A), and results of the peak purity testing for compounds 1–3 (Fig. 2B). In **Fig. 2B**, peak areas where purity was found over 99.9% (green) are represented as mean of percentages ± SD; n = 3.

A quantitative determination of the three main UV active constituents, previously identified as chlorogenic acid (**1**), rutin (**2**) and isoquercitrin (**3**), was performed from the crude, 70% ethanol extract. Three independently measured samples of the extract were analyzed by diode-array detected (DAD) high-pressure liquid chromatography (HPLC). The two UV chromatograms used for the quantitative determination, as well as the results of the peak purity testing for the peaks of compounds **1**–**3** are presented in Figure 2.

Peak purity testing is a built-in feature of the chromatographic software ChromNAV, and is performed on the basis of a systematic comparison of the UV spectra in a previously defined wavelength range within the peak. This gives a purity distribution map that can be displayed graphically as shown in Figure 2B. As a purity of over 99.9% was found for over 95% of each peak area, and practically all remaining peak areas possessed purities between 99.0 and 99.9% in the selected wavelength ranges, it can be concluded that very good detection selectivities were achieved for the three compounds of interest.

**Figure 3:**
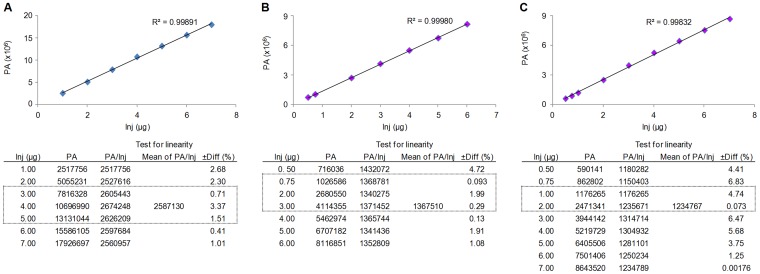
Calibration curves and corresponding data for the three compounds of interest, chlorogenic acid (Fig. 3A), rutin (Fig. 3B) and isoquercitrin (Fig. 3C). Inj: injected amount, PA: peak area, ±Diff(%): difference between the actual value of PA/inj and the averages of each such value in the table, which difference should be less than 5%. Dashed frames in each data table represent ranges where corresponding peak areas of the extracts were found when tested.

Calibration data for the three compounds are shown in Figure 3. For chlorogenic acid and rutin, a very good linearity of the calibration curves was found. Linearity was also tested using strict statistical criteria at all data points, by evaluating the differences between the mean and the actual values of peak area divided by the sample amount. In case of a very good linearity these difference values should not be higher than 5% for any of the data points. In case of isoquercetrin, three out of nine data points showed differences in the range of 5.68–6.83%, when the mean and the actual values of peak area divided by the sample amount were compared, which still represents an acceptable linearity of the calibration curve. Therefore, injected amounts of the three samples to be analyzed were chosen in a way that each peak area of compounds **1**–**3** falls in the region of its calibration curve, where data points passed the linearity testing criteria (*i.e.* less than 5% difference). Dashed frames in Figure 3C illustrate the ranges of the calibration points where each compound of interest was determined.

Calculated amounts and standard deviations for the three compounds of interest present in the extract were as follows: 3.58±0.06% for chlorogenic acid, 1.96±0.03% for rutin and 1.20±0.02% for isoquercitrin.

The animal experiments were designed based on the results of the quantitative analysis: anti-diabetic activity of the mulberry leaf extract was tested in doses of 250 and 750 mg/kg, and compounds **1**–**3** were tested in doses corresponding to the amounts present in such doses of the extract. Activity testing of a mixture of the three compounds in a ratio corresponding to that present in the extract was additionally attempted. Unfortunately, this combination study did not lead to any conclusive results; hence it is not discussed here.

Blood samples were taken on the 4^th^, 8^th^ and 11^th^ days; results are detailed in Table 1.

**Table 1:** Plasma glucose levels in the different treatment groups.

Groups	Day 0	Day 4	Day 8	Day 11
Control	6.32±0.41	4.98±0.33	4.92±0.17	5.41±0.17
Glibenclamide	5.52±0.39	4.36±0.29	4.57±0.12	4.62±0.26*^(P)^
MA (250 mg/kg)	7.31±0.80	5.32±0.17	5.36±0.36	4.37±0.24*
MA (750 mg/kg)	5.60±0.27	5.17±0.25	5.41±0.30	4.17±0.22**
**1** (9 mg/kg)	6.08±0.48	4.80±0.33	4.97±0.42	5.03±0.20
**1** (27 mg/kg)	6.11±0.41	5.03±0.26	4.52±0.37	4.61±0.23*^(P)^
**2** (5 mg/kg)	6.79±1.41	5.27±0.45	5.64±0.32	4.71±0.16*^(P)^
**2** (15 mg/kg)	6.36±0.39	5.33±0.22	5.90±0.21	4.65±0.12*^(P)^
**3** (3 mg/kg)	6.48±0.81	5.13±0.13	5.76±0.49	4.82±0.23
**3** (9 mg/kg)	6.64±0.67	5.57±0.34	5.91±0.32	5.10±0.37

Results are shown as mean ± SEM; Control: 0.25% of methylcellulose, MA: *Morus alba* leaf extract, **1**: chlorogenic acid, **2**: rutin, **3**: isoquercitrin; * and **: p<0.05 and 0.01, respectively by one-way ANOVA followed by Dunnett's multiple comparison test, *^(P)^: p<0.05 by one-way ANOVA followed by Bonferroni post test with uncorrected P value and confidence interval, as compared to the control group.

**Figure 4:**
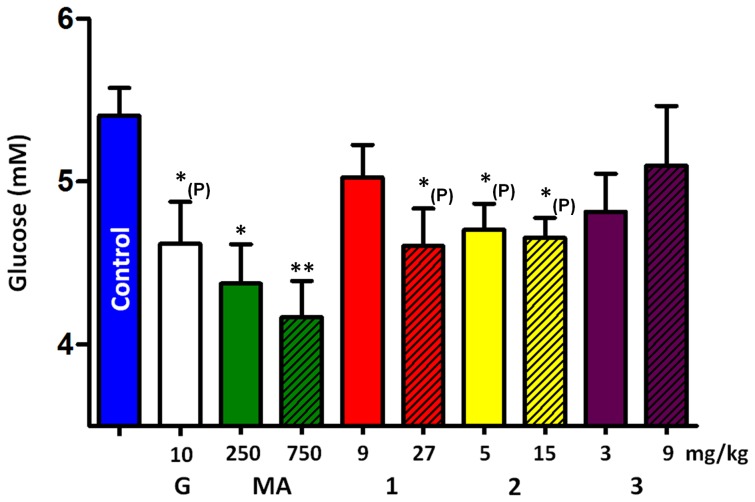
Plasma glucose levels after 11 days of treatment, where significant differences to the control group were found. Results are shown as mean ± SEM, G: glibenclamide; for further details see Table 1 legend.

As a first approach, statistical analysis of the results was performed by one-way ANOVA with Dunnett's multiple comparison test, and statistically significant activities were found after 11 days of treatment. In order to decrease the possibility of false negative results, further, planned comparisons between the treated groups' datasets and that of the control group were also performed by using one-way ANOVA with separate Bonferroni post tests utilizing uncorrected P values and confidence intervals. These results are outlined in Figure 4.

After 11 days of treatment, the extract showed a significant, dose related anti-diabetic activity on our *in vivo* model of type II diabetes. By means of planned comparisons, chlorogenic acid and rutin also showed significant anti-diabetic activity with tendencies for dose dependency, which was not the case for isoquercitrin. Since no fasting periods were scheduled for the animals, the determined glucose levels indicate random plasma glucose values and therefore they are closer to the represented human situation, i.e. not insulin dependent diabetes mellitus. Moreover, based on the activity data, chlorogenic acid and rutin content seems to be responsible for approximately half of the activity of the tested extract in our experimental model. This finding is remarkable considering the richness of mulberry leaves in various anti-diabetic constituents, and it also suggests that these two compounds are excellent markers for a simple and convenient UV-detection based standardization procedure of anti-diabetic products prepared from this drug. Nevertheless, we can also conclude that the α-glucosidase activating effect of the flavonoid fraction previously obtained from mulberry leaves [19] has, at least in case of rutin, less biological significance than other mechanisms of action that together lead to an anti-diabetic effect *in vivo*. On the other hand, other flavonoids present in the drug might still have the potential to exert a blood sugar level increasing activity via an increase of glucose absorption. This would also mean, that in case of a well-designed mulberry preparation for anti-diabetic purposes the high chlorogenic acid and rutin content should be accompanied by low levels of certain undesired flavonoid(s) – future research is needed to clarify whether such criteria are necessary or not.

## Conclusions

Our results can briefly be summarized as follows.

1. A significant, dose-dependent anti-diabetic activity was found for the 70% aqueous ethanolic extract of *Morus alba* leaves on our *in vivo* model of type II. diabetic rats.
2. An analitical method was developed for the rapid, selective determination of three, potentially active, major constituents (chlorogenic acid, rutin and isoquercitrin) of the extract by HPLC-DAD.
3. Contribution of the three major constituents to the overall activity was investigated, and a dose related activity was observed for chlorogenic acid and rutin but not for isoquercitrin. The two previous compounds were found to play an important role in the anti-diabetic effect of mulberry leaves: *ca.* half of the observed activity can apparently be explained by their presence. Although testing the three compounds was also attempted in combination, at this time no conclusion on the presence or absence of synergistic effect can be made.
4. Based on the above, our analytical method can provide a valuable tool and a reasonable alternative of the existing methods for the quality control of mulberry products.

## Materials and Methods

### Ethics statement

The animals were treated in accordance with the European Communities Council Directives (86/609/ECC). The experimental animal protocol satisfied the Guidelines for Animal Experimentation approved by the Animal Experimentation Committee of the University of Szeged (approval no: IV/01758–1/2008). Rats were kept at 22 3°C; the relative humidity was 30–70% and maintained on a 12 h light:12 h darkness cycle. The animals were maintained on a standard rodent pellet diet (Charles-River Laboratories, Isaszeg, Hungary) with tap water available *ad libitum*. After the experiments, they were sacrificed by CO_2_ inhalation. Field activity for collecting plant sample did not require a specific permission, the location was neither privately-owned nor protected in anyway, and no protected or endangered species were involved.

### Plant material & Extraction

*Morus alba* leaves were collected near Ásotthalom, Hungary in August, 2009. Botanical identification was done by A. Hunyadi, and a voucher specimen (collection No. MA082009) was deposited in the Institute of Pharmacognosy, University of Szeged, Szeged, Hungary. Two kg of air-dried leaves were extracted with 70% aqueous ethanol and evaporated to dryness under reduced pressure at 50°C to yield 675.4 g of dried extract (MA).

### Solvents and standards

Chromatographic solvents were purchased from Merck Chemicals (Budapest, Hungary). Chlorogenic acid, rutin and isoquercitrin were purchased from Sigma-Aldrich (Budapest, Hungary), ChromaDex (Irvine, CA, USA) and Extrasynthése (Genay, France), respectively. These materials were used both as standards for the quantitative determination and as pure compounds tested in the bioassay.

### HPLC analysis

A quantitative determination was performed for the main UV-active and potentially anti-diabetic constituents, chlorogenic acid, rutin and isoquercitrin by HPLC on a gradient system of two Jasco PU2080 pumps connected to a Jasco MD-2010 Plus diode-array detector (DAD). A Zorbax SB C-18 (5 µm, 4.6×250 mm) column was used with an aqueous acetonitrile gradient (10% smoothly increasing to 23 in 17 min, keeping the same ratio till t = 25 min and returning to the original composition) at a flow rate of 1 mL/min. Both ACN and water contained 0.05% of TFA. DAD data were collected from 210 to 400 nm, and each compound was determined at its UV absorbance maximum: λ = 326.3 nm (chlorogenic acid) and λ = 353.0 nm (rutin and isoquercitrin). Three independently measured samples were analyzed (10.0, 10.2 and 11.9 mg), each dissolved in 1.00 mL of 50% MeOH, and 10 µL of solution was injected.

In order to investigate detection selectivity, peak purity tests were also performed by systematically comparing all obtained UV spectra between λ = 300–400 nm (chlorogenic acid) and λ = 350–450 nm (rutin and isoquercitrin) within the peaks of interest.

Calibration curves were obtained from analyzing 10 µL of 0.1–0.7 (7 data points), 0.05–0.6 (7 data points) and 0.05–0.7 (9 data points) mg/mL solutions of chlorogenic acid, rutin and isoquercitrin, respectively. Standard linear regression was used for calculating the regression coefficient (R^2^), and linearity of each calibration curve was also tested by calculating the differences between each peak area (PA) divided by the amount of analyte injected and the mean of these values within the dataset. Linearity was accepted when differences were found to be less than ±5%.

### *In vivo* bioassay

Newborn Sprague-Dawley rats (two days after birth) were treated with 150 mg/kg streptozotocin intraperitoneally. The animals were housed in plastic cages in a thermoneutral environment (22±1°C). Eight weeks later, the animals were randomized into different treatment groups (n = 6 or 7). Once a day, the different groups were orally treated with extract (250 or 750 mg/kg), chlorogenic acid (9 or 27 mg/kg), rutin (5 or 15 mg/kg) or isoquercitrin (3 or 9 mg/kg) suspended in 0.25% of methylcellulose; the dosing volume was 5 mL/kg for all treatments. Standard food and tap water were freely available. Venous blood samples from the tail were collected and plasma glucose concentration was determined by means of glucose oxidase – peroxidase colorimetric method on the 4^th^, 8^th^ and 11^th^ days of the treatment (Reanal Finechamical Co, Budapest, Hungary). All the calculations and statistical evaluation were performed by GraphPad Prism 5 (GraphPad Software, San Diego, CA, USA).
